# Long non-coding RNA MALAT1 enhances the apoptosis of cardiomyocytes through autophagy inhibition by regulating TSC2-mTOR signaling

**DOI:** 10.1186/s40659-019-0265-0

**Published:** 2019-11-29

**Authors:** Hao Hu, Jiawei Wu, Xiaofan Yu, Junling Zhou, Hua Yu, Likun Ma

**Affiliations:** Department of Cardiovascular, The First Affiliated Hospital of USTC, Division of Life Sciences and Medicine, University of Science and Technology of China, No. 1 Swan Lake Road, Hefei, 230001 Anhui People’s Republic of China

**Keywords:** MALAT1, Cardiomyocyte apoptosis, Autophagy, TSC2-mTOR, EZH2

## Abstract

**Background:**

Our previous study showed that knockdown of long noncoding RNA (lncRNA) metastasis-associated lung adenocarcinoma transcript 1 (MALAT1) attenuated myocardial apoptosis in mouse acute myocardial infarction (AMI). This study aims to explore whether MALAT1 enhanced cardiomyocyte apoptosis via autophagy regulation and the underlying mechanisms of MALAT1 regulating autophagy.

**Methods:**

Cardiomyocytes were isolated from neonatal mice and then stimulated with hypoxia/reoxygenation (H/R) injury to mimic AMI. The autophagy level was assessed using GFP-LC3 immunofluorescence and western blot analysis of autophagy-related proteins. RNA pull-down and RNA immunoprecipitation (RIP) was performed to analyze the binding of MALAT1 and EZH2. Chromatin immunoprecipitation (ChIP) assay was performed to analyze the binding of TSC2 promoter and EZH2. The cell apoptosis was evaluated using TUNEL staining and western blot analysis of apoptosis-related proteins.

**Results:**

H/R injury increased MALAT1 expression in cardiomyocytes. Furthermore, MALAT1 overexpression inhibited, whereas MALAT1 knockdown enhanced the autophagy of cardiomyocytes. Moreover, MALAT1 overexpression recruited EZH2 to TSC2 promoter regions to elevate H3K27me3 and epigenetically inhibited TSC2 transcription. Importantly, TSC2 overexpression suppressed mTOR signaling and then activated the autophagy. Further results showed that MALAT1 inhibited proliferation and enhanced apoptosis of cardiomyocytes through inhibiting TSC2 and autophagy.

**Conclusion:**

These findings demonstrate that the increased MALAT1 expression induced by H/R injury enhances cardiomyocyte apoptosis through autophagy inhibition by regulating TSC2-mTOR signaling.

## Background

Acute myocardial infarction (AMI) is the most serious cardiovascular disease with high morbidity and mortality worldwide [1]. Inhibition of myocardial apoptosis has been regarded as a pivotal therapeutic target for AMI [2]. Long non-coding RNAs (lncRNAs) are transcripts of more than 200 nucleotides in length without coding potential, which play significant roles in the pathogenesis of various human diseases, including AMI [3, 4]. Mounting evidence suggests that lncRNA metastasis-associated lung adenocarcinoma transcript 1 (MALAT1) plays important roles in the pathogenesis and development of various human diseases [5–7]. Our previous study has confirmed that knockdown of MALAT1 attenuated myocardial apoptosis in mouse AMI [8]. However, the mechanisms by which MALAT1 enhanced cardiomyocyte apoptosis remain not fully understood.

Autophagy is an evolutionarily conserved intracellular degradation process by which cytoplasmic constituents are delivered to the lysosome for digestion [9]. Studies show that autophagy plays distinct roles in the occurrence and development of AMI [2]. Inhibition of autophagy has been shown to aggravate the ischemia-induced myocardial damage [10]. Mammalian target of rapamycin (mTOR) is a negative regulator of autophagy [11, 12]. Evidence suggests that tuberous sclerosis 2 (TSC2) suppresses mTOR signaling and thus induces autophagy [13, 14]. However, there is no report covering the role of TSC2-mTOR-autophagy signaling in AMI.

EZH2 (enhancer of zeste 2 polycomb repressive complex 2 subunit) has histone methyltransferase activity with substrate specificity for catalyzing tri-methylation of the 27th lysine of H3 in nucleosome histone (H3K27me3), which is a repressive histone mark associated with gene repression [15]. Several reporters have demonstrated that MALAT1 can directly bind to methyltransferase EZH2 and regulate expression of downstream genes by recruiting EZH2 [16, 17]. In addition, EZH2 has been shown to epigenetically repress TSC2 [18]. Furthermore, the downregulation of TSC2 by EZH2 elicited mTOR activation, which in turn modulated subsequent mTOR pathway-related events, including inhibition of autophagy [18]. These findings implied that MALAT1 might epigenetically repress TSC2 transcription via recruiting EZH2 to TSC2 promoter regions, and thereby induce mTOR activation and subsequent autophagy inhibition.

In this study, we investigated the role of TSC2-mTOR-autophagy signaling in the MALAT1-mediated attenuation of myocardial apoptosis. Furthermore, we elucidated whether MALAT1 epigenetically repress TSC2 transcription via recruiting EZH2 to TSC2 promoter regions.

## Materials and methods

### Cell culture and isolation

Cardiomyocytes were isolated from neonatal mice as previously described [8]. Briefly, the dissected hearts were rinsed in HEPES-buffered saline solution, cut into pieces, and digested with 0.25% trypsin. Afterwards, the cells were maintained in Dulbecco’s modified Eagle medium/F-12 (DMEM/F12; Gibco, Carlsbad, CA, USA) supplemented with 5% fetal bovine serum (FBS; Gibco), 0.1 mM ascorbate, insulin-transferring-sodium selenite media supplement (Sigma-Aldrich, St. Louis, MO, USA), 100 U/ml penicillin, 100 μg/ml streptomycin, and 0.1 mM bromodeoxyuridine (Gibco) in an atmosphere containing 5% CO_2_ at 37 °C.

### Hypoxia/reoxygenation (H/R) injury

Mouse cardiomyocytes were cultured under normoxic condition for 24 h, after which cardiomyocytes were washed twice in PBS and cultured under hypoxia (5% CO_2_, 95% N_2_) for 4 h and then under reoxygenation condition (5% CO_2_, 95% O_2_) for another 4 h to stimulate H/R injury.

### RNA extraction and quantitative real-time PCR (qRT-PCR)

Total RNA was extracted from cells using TRIzol reagent (Invitrogen; Thermo Fisher Scientific, Inc., Waltham, MA, USA). RNA was reverse transcribed into cDNAs using the Reverse Transcription Kit (TaKaRa, Dalian, China). The cDNA template was synthesized through qRT-PCR using SYBR Green PCR Kit (Thermo Fisher Scientific, Inc.) by the ABI7500 system (Applied Biosystem, Foster City, CA, USA). The relative expression of MALAT1 and HIF-1α were normalized to the internal control GAPDH using the 2^−ΔΔCt^ method.

### Western blot

Total protein was extracted from mouse cardiomyocytes using the RIPA Lysis Buffer (Beyotime, Shanghai, China). The proteins were separated by 10% SDS-PAGE gels and electro-transferred onto a PVDF membrane (Millipore, Billerica, MA, USA). Subsequently, the membranes were blocked with 5% non-fat dried milk and then incubated with the following primary antibodies against HIF-1α (1:1000; Abcam, Cambridge, MA, USA), LC3-I and LC3-II (both from Anti-LC3B antibodies, 1:1000, Sigma-Aldrich), Beclin-1 (1:1000, Abcam), p-mTOR (1:1000, Santa Cruz Biotechnology, Dallas, TX, USA), mTOR (1:1000, Santa Cruz Biotechnology), TSC2 (1:1000; Cell Signaling Technology, Danvers, MA, USA), H3K27me3 (1:1000; Cell Signaling Technology), EZH2 (1:1000; Cell Signaling Technology), Caspase-3 (1:1000, Abcam), Bax (1:500, Santa Cruz Biotechnology), Bcl-2 (1:500, Santa Cruz Biotechnology), followed by horseradish peroxidase (HRP)-coupled secondary antibodies. The protein was detected with an enhanced chemiluminescence kit (Applygen Technologies, Beijing, China). β-tubulin or β-actin served as the loading control.

### Green fluorescent protein (GFP)-LC3 immunofluorescence

GFP-LC3 immunofluorescence was performed as previously described [19]. GFP-LC3 transfection was performed following the manufacturer’s instructions (Genomeditech, Shanghai, China). LC3 protein was indicated by the aggregated green fluorescent particles. Fluorescence images were captured and analyzed under a fluorescence microscope (Nikon Corporation, Tokyo, Japan).

### Cell transfection

The pcDNA3.1-MALAT1, pcDNA3.1-EZH2, pcDNA3.1-TSC2, MALAT1 siRNA (si-MALAT1), si-EZH2, and their corresponding controls were purchased from Shanghai GenePharma Co., Ltd. (Shanghai, China). Mouse cardiomyocytes were seeded into 24-well plates and transfected with oligonucleotides or plasmids using Lipofectamine 2000 (Invitrogen) following the manufacturer’s instructions.

### RNA pull-down assay

The interaction between MALAT1 and EZH2 protein was analyzed using the Pierce™ Magnetic RNA–Protein Pull-Down Kit (Thermo Fisher Scientific, Inc.) following the manufacturer’s instructions. The RNA-binding protein complexes were washed, eluted, and analyzed using western blot analysis.

### RNA immunoprecipitation (RIP)

The binding between MALAT1 and EZH2 was examined using the RNA-Binding Protein Immunoprecipitation Kit (EMD Millipore, USA) following the manufacturer’s instructions. The cells were lysed and the cell lysis solutions were incubated with antibody against EZH2 or isotype control IgG. The RNA–protein complexes were immunoprecipitated with protein A agarose beads and RNA was extracted using TRIzol (Invitrogen). MALAT1 was quantified using qRT-PCR.

### Chromatin immunoprecipitation (ChIP) assay

The ChIP assay was performed using a ChIP Assay Kit (EMD Millipore, USA) according to the manufacturer’s instructions. The resulting solutions were incubated with anti-H3K27me3 antibody, or IgG (Cell Signaling Technology) and DNA was purified using the QIAquick PCR Purification Kit (Qiagen, USA). qRT-PCR was performed to quantify the precipitated TSC2 expression level.

### Determination of LDH release

The cell supernatant was collected and centrifuged at 4 °C for 10 min. The supernatant was collected and prepared for determination of LDH release using a LDH assay kit (Nanjing Jiancheng Bioengineering Institute, Nanjing, China) according to the manufacturer’s instructions.

### Cell apoptosis assay

The cell apoptosis was analyzed using One Step TUNEL Apoptosis Assay Kit (Beyotime). The images of the FITC-labeled TUNEL-positive cells were captured using a fluorescent microscope (Nikon Corporation) according to the manufacturer’s instructions. The cell nucleus was labeled in blue by DAPI (Invitrogen). The nick-ends labeled in green indicated the apoptotic cells.

### Cell viability assay

Cell viability assay was conducted using the MTT assay. Briefly, mouse cardiomyocytes were seeded in the 96‐well plates and were given different treatments, 20 μl MTT (Sigma-Aldrich) was added to a final concentration of 0.5 mg/ml, and the cells were incubated for 4 h at 37 °C. Then the medium was replaced with 150 μl DMSO for 10 min. The absorbance at 490 nm was measured using the Fluoroskan Ascent Fluorometer (Thermo Fisher Scientific, Helsinki, Finland).

### Statistical analysis

All statistical analyses were performed using SPSS 16.0. The data are presented as the mean ± standard deviation (SD). The differences between groups were analyzed using the unpaired Student’s *t* test for two groups and one-way analysis of variance (ANOVA) for three or more groups. p < 0.05 was considered statistically significant.

## Results

### H/R injury increased MALAT1 expression and enhanced the autophagy in cardiomyocytes

**Fig. 1:**
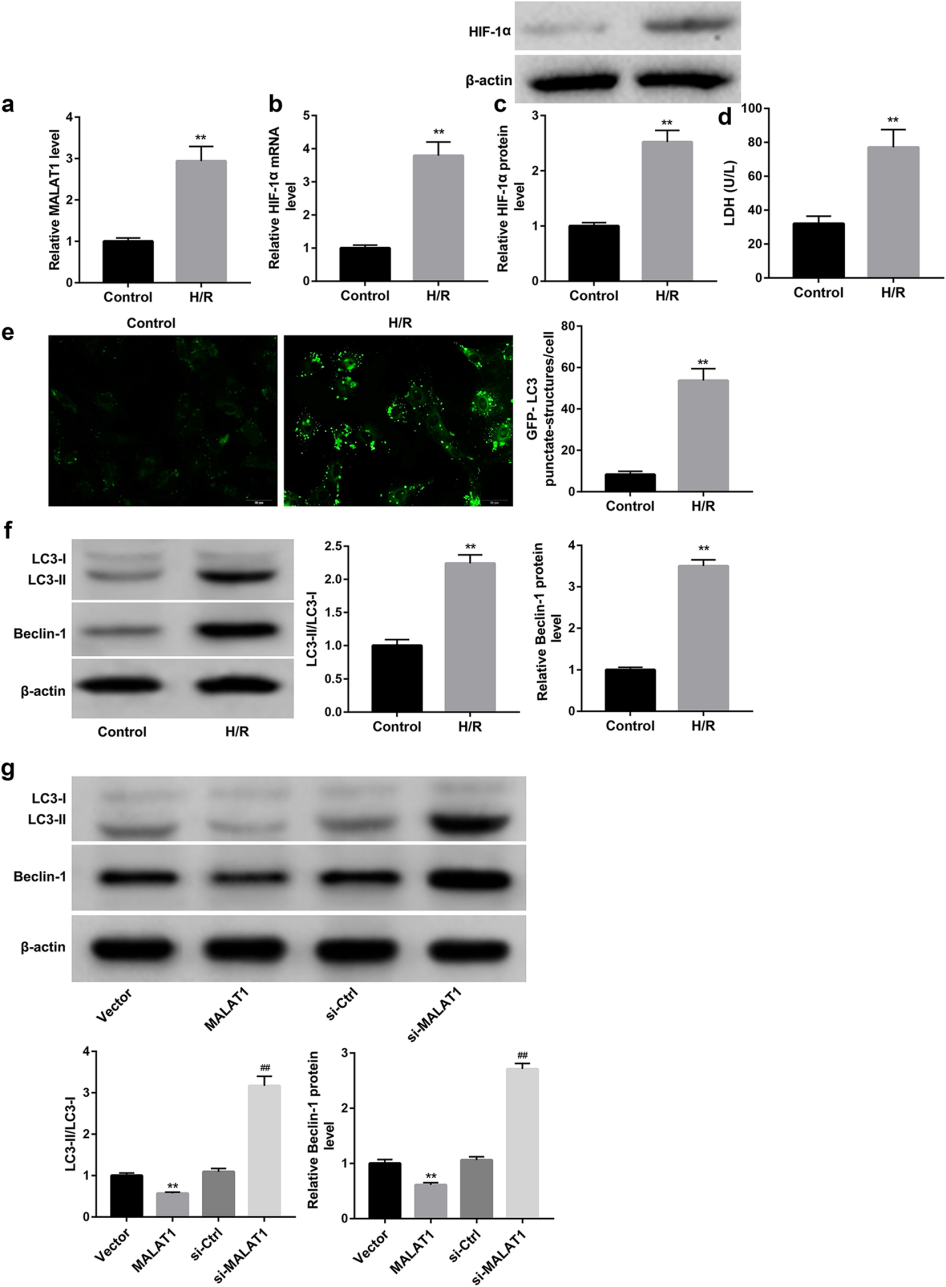
MALAT1 overexpression inhibited, whereas MALAT1 knockdown enhanced the autophagy of cardiomyocytes stimulated with H/R injury. Cardiomyocytes were isolated from neonatal mice and then stimulated with H/R injury. qRT-PCR analysis of relative MALAT1 level (**a)** and HIF-1α mRNA level (**b)** in cardiomyocytes. **c** Western blot analysis of HIF-1α protein level in cardiomyocytes. **d** LDH release in cardiomyocytes. **e** The autophagosome puncta of GFP-LC3 by immunofluorescence in cardiomyocytes. Scale bar: 20 μm. **f** Western blot was performed to examine the protein levels of LC3-I, LC3-II, and Beclin-1 in cardiomyocytes. **g** Western blot was performed to examine the protein levels of LC3-I, LC3-II, and Beclin-1 in cardiomyocytes which were transfected with pcDNA3.1-MALAT1 (MALAT1), empty pcDNA3.1 (Vector), si-MALAT1, or scramble siRNA (si-Ctrl), followed by stimulation with H/R injury. Their quantitative analysis was normalized to β-actin. **a**–**f** **p < 0.01 vs. Control group. **g** **p < 0.01 vs. Vector group, ^##^p < 0.01 vs. si-Ctrl group

The mouse cardiomyocytes following H/R injury showed significantly higher MALAT1 level compared with the control cardiomyocytes (Fig. 1a). Furthermore, data revealed that the mRNA and protein levels of hypoxia inducible factor-1α (HIF-1α) were both greatly induced following H/R injury (Fig. 1b, c). Moreover, release of lactate dehydrogenase (LDH) which indicated the injury of cardiomyocytes was significantly induced by H/R injury (Fig. 1d). We also found that the mouse cardiomyocytes following H/R injury exhibited stronger GFP-LC3 puncta and increased percentage of GFP-LC3 cells when compared with the control cardiomyocytes (Fig. 1e). Additionally, the mouse cardiomyocytes following H/R injury also showed increased protein levels of autophagy molecular markers including Beclin-1 and LC3-II, as well as the LC3-II/LC3-I ratio when compared with control group (Fig. 1f). These data indicated that H/R injury enhanced the autophagy of cardiomyocytes.

### MALAT1 overexpression inhibited, whereas MALAT1 knockdown enhanced the autophagy of cardiomyocytes

We next evaluated the role of MALAT1 in the H/R injury-induced autophagy. The results showed that MALAT1 overexpression significantly decreased, whereas MALAT1 knockdown increased protein levels of Beclin-1 and LC3-II, as well as the ratio of LC3-II/LC3-I (Fig. 1g). These results indicated that MALAT1 suppressed the H/R injury-induced autophagy of cardiomyocytes.

### MALAT1 overexpression recruited EZH2 to elevate H3K27me3 and epigenetically inhibited TSC2

**Fig. 2:**
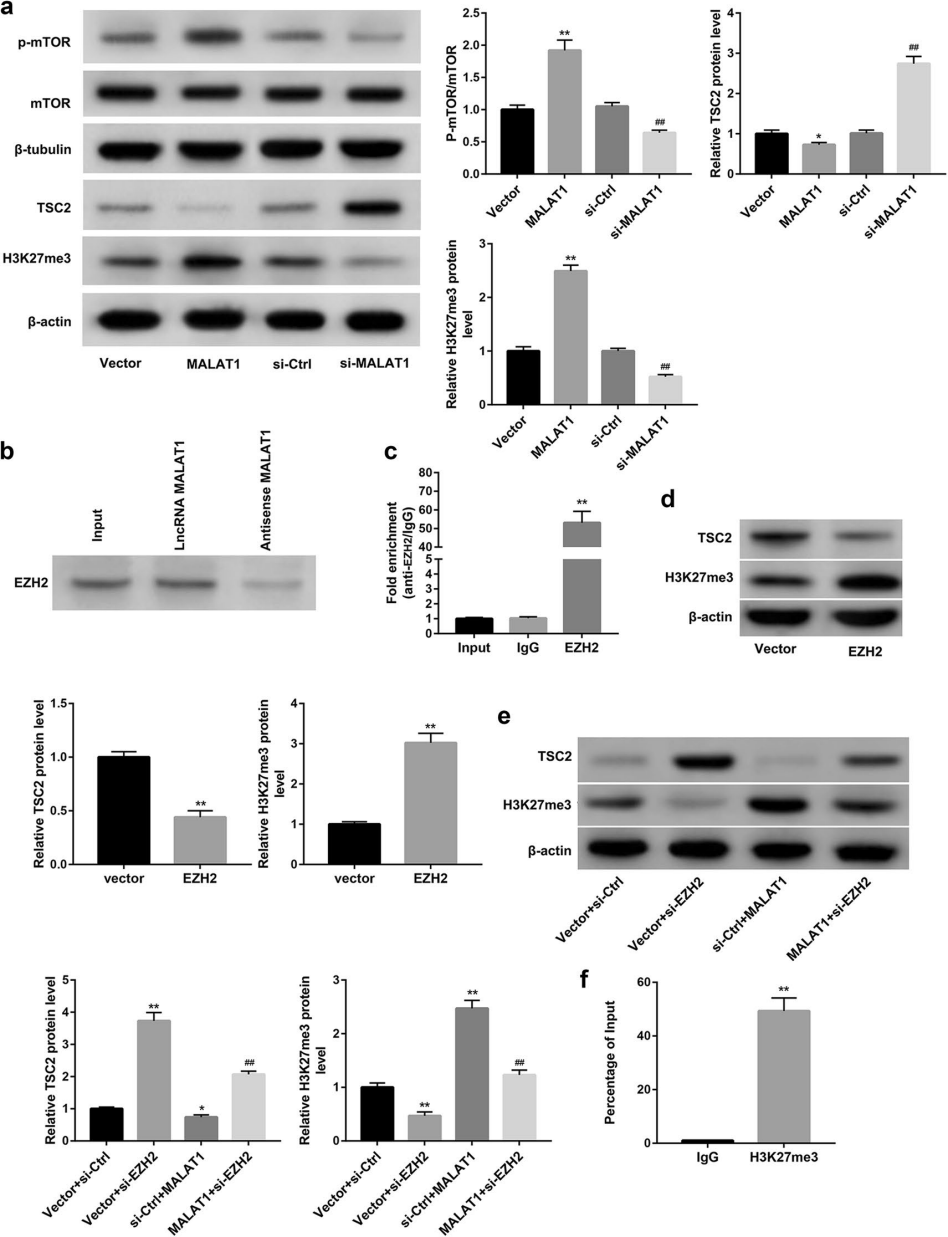
MALAT1 overexpression recruited EZH2 to elevate H3K27me3 and epigenetically inhibited TSC2 expression. Cardiomyocytes were transfected with pcDNA3.1-MALAT1 (MALAT1), empty pcDNA3.1 (Vector), si-MALAT1, or scramble siRNA (si-Ctrl), followed by stimulation with H/R injury. **a** Western blot was performed to measure the protein levels of TSC2, p-mTOR, and H3K27me3. *p < 0.05, **p < 0.01 vs. Vector group, ^##^p < 0.01 vs. si-Ctrl group. **b** RNA pull-down and **c** RIP was performed to analyze the binding of MALAT1 and EZH2. **p < 0.01 vs. IgG group. **d** Western blot was performed to detect the protein levels of TSC2 and H3K27me3 in cardiomyocytes transfected with pcDNA3.1-EZH2 (EZH2) or empty pcDNA3.1 (Vector). **p < 0.01 vs. Vector group. **e**, **f** Cardiomyocytes were co-transfected with pcDNA3.1-MALAT1 (MALAT1) or empty pcDNA3.1 (Vector), with si-EZH2 or si-Ctrl. **e** Western blot was performed to detect the protein levels of TSC2 and H3K27me3. *p < 0.05, **p < 0.01 vs. Vector + si-Ctrl group, ^##^p < 0.01 vs. si-Ctrl + MALAT1 group. **f** CHIP experiments displayed that TSC2 promoter was enriched with H3K27me3. ^**^p < 0.01 vs. IgG group

We next explored the mechanism by which MALAT1 overexpression inhibited autophagy. Evidence suggests that TSC2 suppresses mTOR signaling and thus induces autophagy [13, 14]. Our results showed that MALAT1 overexpression decreased protein levels of TSC2, whereas increased phosphorylation level of mTOR (p-mTOR). In contrast, MALAT1 knockdown exerted the opposite effect (Fig. 2a).

We then investigated the mechanism whereby MALAT1 inhibited TSC2 protein level. Studies show that EZH2 has histone methyltransferase activity with substrate specificity for catalyzing H3K27me3, a repressive histone mark associated with gene repression. Our data from the RNA pull-down (Fig. 2b) and RIP (Fig. 2c) confirmed the binding of MALAT1 to EZH2. Furthermore, MALAT1 overexpression significantly increased, whereas MALAT1 knockdown decreased protein levels of H3K27me3 (Fig. 2a). Moreover, similar with effect of MALAT1 overexpression, EZH2 overexpression decreased protein levels of TSC2, whereas increased protein levels of H3K27me3 (Fig. 2d). To further determine whether MALAT1 decreased TSC2 and increased H3K27me3 via recruiting EZH2, we co-transfected cardiomyocytes with pcDNA3.1-MALAT1 and si-EZH2. Data revealed that EZH2 knockdown significantly increased protein levels of TSC2 and decreased protein levels of H3K27me3. More importantly, EZH2 knockdown effectively abrogated the MALAT1 overexpression-mediated decrease in TSC2 and increase in H3K27me3 protein levels (Fig. 2e). Moreover, results of ChIP confirmed that TSC2 promoter was enriched with H3K27me3 (Fig. 2f). Collectively, these results indicated that MALAT1 overexpression recruited EZH2 to elevate H3K27me3 and epigenetically inhibited TSC2.

### MALAT1 overexpression suppressed the autophagy of cardiomyocytes through inhibiting TSC2-mTOR signaling

**Fig. 3:**
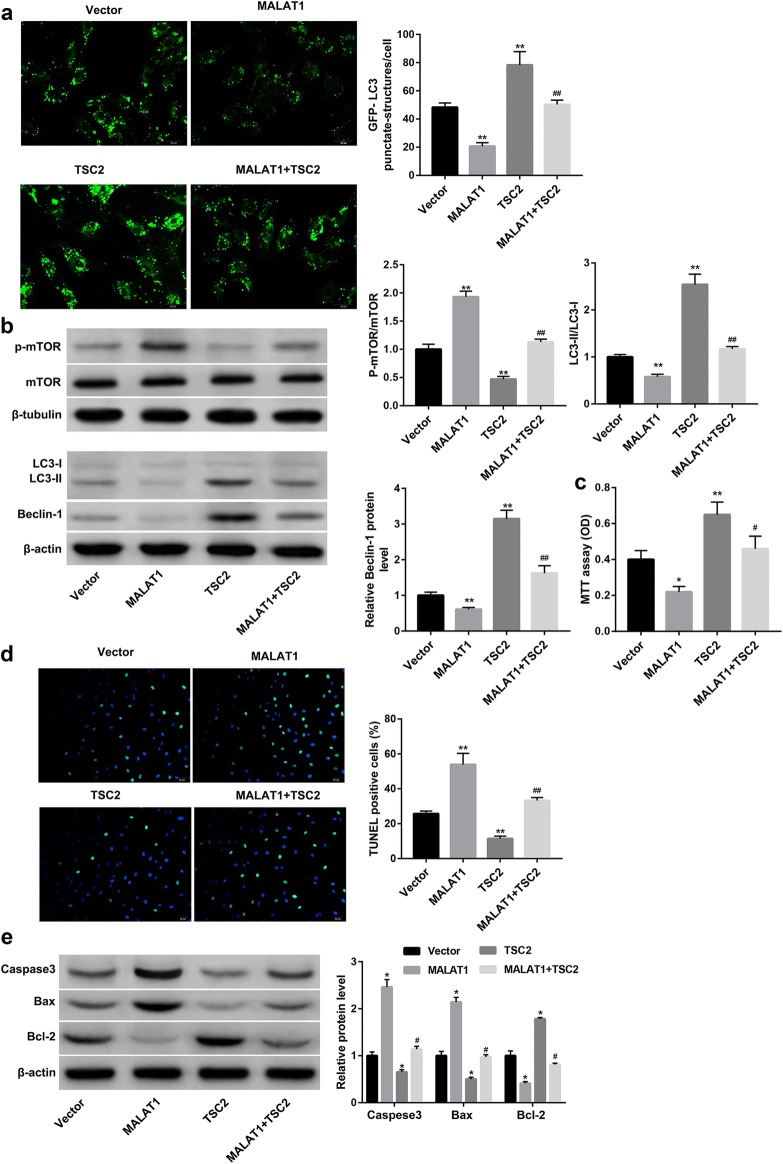
MALAT1 inhibited autophagy and enhanced apoptosis of cardiomyocytes through inhibiting TSC2. Cardiomyocytes were co-transfected with pcDNA3.1-MALAT1 (MALAT1) or empty pcDNA3.1 (Vector), and pcDNA3.1-TSC2 (TSC2) or Vector, followed by stimulation with H/R injury. **a** The autophagosome puncta of GFP-LC3 by immunofluorescence in cardiomyocytes. Scale bar: 20 μm. **b** Western blot was performed to examine the protein levels of p-mTOR, LC3-I, LC3-II, and Beclin-1 in cardiomyocytes. **c** The cell proliferation was evaluated using MTT assay. **d** The cell apoptosis was evaluated using TUNEL staining. The nick-ends were labeled in green indicating the apoptotic cells and the cell nucleus was labeled in blue by DAPI. Scale bar: 20 μm. **e** Western blot was performed to detect protein expression of Caspase-3, Bax, and Bcl-2 in cardiomyocytes. *p < 0.05, **p < 0.01 vs. Vector group; ^#^p < 0.05, ^##^p < 0.01 vs. MALAT1 group

To further verify whether MALAT1 suppressed autophagy through inhibiting TSC2, cardiomyocytes were co-transfected with pcDNA3.1-MALAT1 and pcDNA3.1-TSC2. Data revealed that, in contrast with MALAT1 overexpression, TSC2 overexpression significantly decreased the phosphorylation level of mTOR, whereas increased the percentage of GFP-LC3 cells (Fig. 3a) and protein levels of Beclin-1 and LC3-II, as well as the LC3-II/LC3-I ratio (Fig. 3b) when compared with the Vector group. The mTOR signaling has been shown to negatively regulate autophagy. Thus, these results confirmed that TSC2 suppressed mTOR signaling and thus induced autophagy.

More importantly, TSC2 overexpression efficiently abolished the MALAT1 overexpression-mediated increase in the phosphorylation level of mTOR, and decrease in the percentage of GFP-LC3 cells (Fig. 3a) and protein levels of Beclin-1 and LC3-II, as well as the LC3-II/LC3-I ratio (Fig. 3b). Together, these data indicated that MALAT1 suppressed autophagy, at least partially, through inhibiting TSC2-mTOR signaling.

### MALAT1 overexpression inhibited proliferation and enhanced apoptosis of cardiomyocytes through inhibiting TSC2

We next examined the role of MALAT1 in regulating the proliferation and apoptosis of cardiomyocytes. As shown in Fig. 3c, MALAT1 notably inhibited cell proliferation of cardiomyocytes. Furthermore, MALAT1 overexpression significantly increased the percentage of TUNEL-positive cells (Fig. 3d) and the protein levels of pro-apoptotic proteins including Caspase-3 and Bax, whereas decreased levels of anti-apoptotic Bcl-2 (Fig. 3e), indicating that MALAT1 overexpression enhanced the apoptosis of cardiomyocytes. We then investigated the role of TSC2 in the MALAT1 overexpression-mediated regulation of cardiomyocyte proliferation and apoptosis. Data revealed that TSC2 overexpression significantly facilitated proliferation and inhibited apoptosis of cardiomyocytes and effectively impaired the MALAT1 overexpression-mediated regulation of cardiomyocyte proliferation and apoptosis (Fig. 3c–e). These results indicated that MALAT1 overexpression inhibited proliferation and enhanced apoptosis of cardiomyocyte, at least partially, through inhibiting TSC2.

### MALAT1 overexpression inhibited proliferation and enhanced apoptosis of cardiomyocytes through inhibiting autophagy

**Fig. 4:**
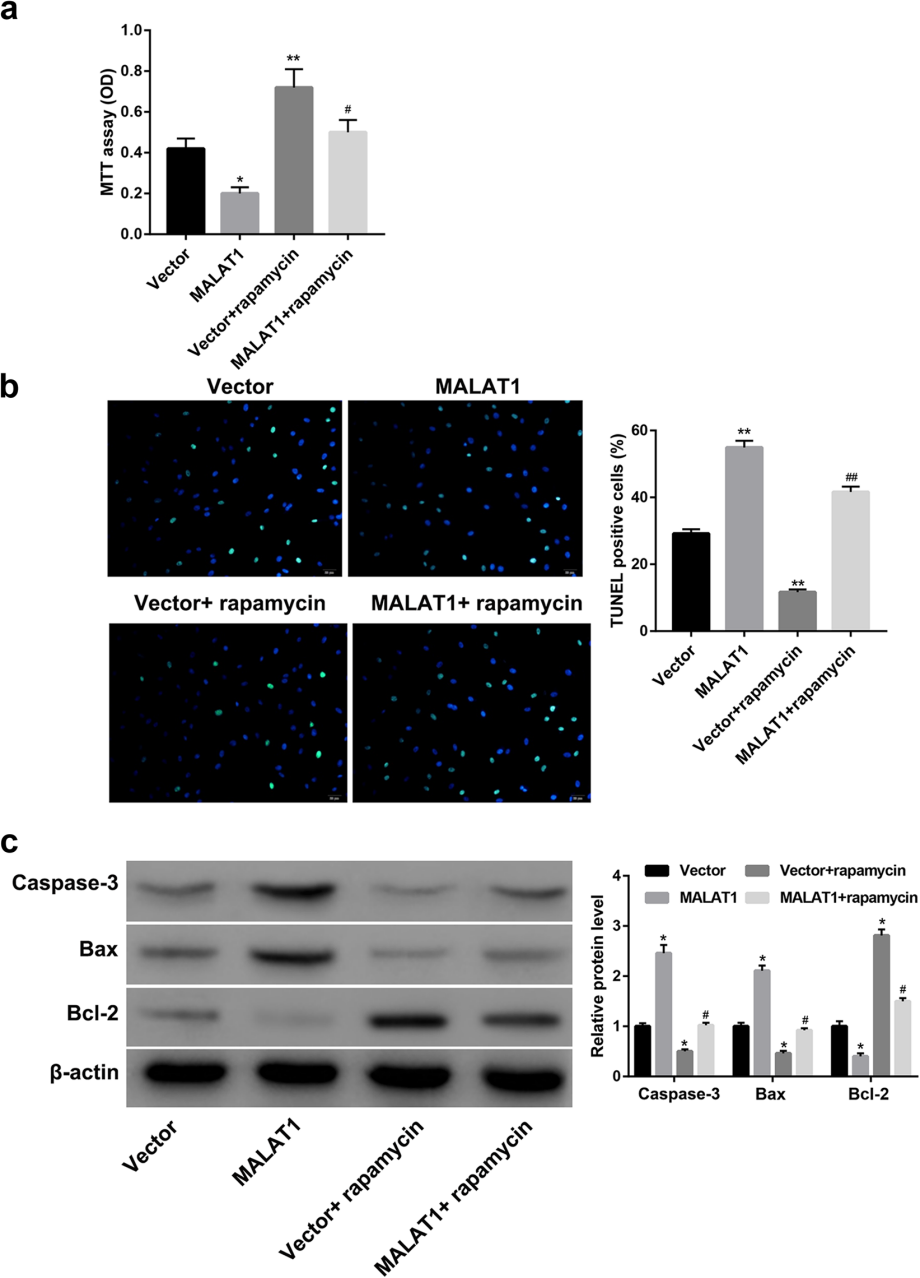
MALAT1 inhibited proliferation and enhanced apoptosis of cardiomyocytes through inhibiting autophagy. Cardiomyocytes were transfected with pcDNA3.1-MALAT1 (MALAT1) or empty pcDNA3.1 (Vector), followed by treatment with autophagy activator rapamycin before stimulation with H/R injury. **a** The cell proliferation was evaluated using MTT assay. **b** The cell apoptosis was evaluated using TUNEL staining. The nick-ends were labeled in green indicating the apoptotic cells and the cell nucleus was labeled in blue by DAPI. Scale bar: 20 μm. **c** Western blot was performed to detect protein levels of Caspase-3, Bax, and Bcl-2 in cardiomyocytes. *p < 0.05, **p < 0.01 vs. Vector group; ^#^p < 0.05, ^##^p < 0.01 vs. MALAT1 group

Finally, we elucidated whether MALAT1 overexpression inhibited proliferation and enhanced apoptosis of cardiomyocytes through autophagy inhibition. To this end, the MALAT1-overexpressing cardiomyocytes were treated with rapamycin (an autophagy activator) before stimulation with H/R injury. Data showed that rapamycin treatment significantly facilitated cell proliferation (Fig. 4a), decreased the percentage of TUNEL-positive cells (Fig. 4b) and protein levels of Caspase-3 and Bax, whereas increased levels of Bcl-2 (Fig. 4c). Importantly, rapamycin treatment effectively rescued the MALAT1 overexpression-mediated effects on cell proliferation (Fig. 4a), percentage of TUNEL-positive cells (Fig. 4b) and protein levels of Caspase3, Bax, and Bcl-2 (Fig. 4c). These results indicated that MALAT1 inhibited proliferation and enhanced apoptosis of cardiomyocytes, at least partially, through autophagy inhibition.

## Discussion

Growing studies indicate that the “adaptive” induction of autophagy promotes cardiomyocyte survival and confers a cardioprotective effect on AMI [20]. Evidence suggests that autophagy can be regulated by certain lncRNAs in human diseases [21, 22]. In this study, we observed that cardiomyocytes increased MALAT1 expression and enhanced autophagy following H/R injury. Furthermore, MALAT1 overexpression inhibited, whereas MALAT1 knockdown enhanced the autophagy of cardiomyocytes. The inhibition of autophagy by MALAT1 in this study was in line with data from a previous study showing that MALAT1 knockdown enhanced the formation of autophagosomes and increased expression of the autophagy-related markers [23].

Studies have demonstrated that TSC2-mTOR signaling plays distinct roles in regulating autophagy [24, 25]. TSC2 protein complexes with TSC1 and blocks the ability of the Rheb (Ras homolog enriched in brain) GTPase to activate mTOR signaling and thus induces autophagy [13, 14]. In addition, the mTOR-autophagy signaling has been involved in regulating the progress of AMI [26]. These results prompt us to investigate whether the mechanism by which MALAT1 inhibited autophagy in H/R-stimulated cardiomyocytes involves its regulation of TSC2-mTOR signaling. Our results showed that TSC2 overexpression effectively rescued the MALAT1 overexpression-mediated inhibition of autophagy and activation of mTOR signaling, indicating that MALAT1 suppressed autophagy via inhibiting TSC2-mTOR signaling. Accordingly, we then explored the mechanism whereby MALAT1 inhibited TSC2-mTOR signaling. Previous data indicated that MALAT1 regulates expression of downstream genes by recruiting methyltransferase EZH2 [16, 17]. In addition, EZH2 has been demonstrated to epigenetically repress TSC2, which in turn elicited mTOR activation and contributed to inhibition of autophagy [18]. However, no literature available on the regulation of TSC2 by MALAT1 has been published. In this study, we provided first evidence that MALAT1 epigenetically repress TSC2 transcription via recruiting EZH2 to TSC2 promoter regions to induce H3K27me3, and thereby induce mTOR activation and subsequent autophagy inhibition.

Autophagy can be activated as a stress response shortly after AMI; however, sustained ischemia impaired cardiomyocyte autophagy flux, which exacerbated the post-infarct adverse cardiac modeling [27]. Our further investigation showed that both TSC2 overexpression and autophagy induction by rapamycin effectively attenuated the MALAT1-mediated regulation of cardiomyocyte proliferation and apoptosis. These data provide evidence that MALAT1 inhibits proliferation and promotes apoptosis of cardiomyocytes, at least partially, through inhibiting the TSC2-mTOR-autophagy signaling. TSC2 acts as an important tumor suppressor gene, mutations within which are associated with the development of tuberous sclerosis and implicated in multiple tumor types [28]. Our results showed that TSC2 overexpression significantly facilitated proliferation and inhibited apoptosis of cardiomyocytes, indicating its potential protective role in AMI.

## Conclusion

In conclusion, the increased MALAT1 expression induced by H/R injury inhibits proliferation and enhances apoptosis of cardiomyocytes through autophagy inhibition by regulating TSC2-mTOR signaling. Furthermore, MALAT1 epigenetically repress TSC2 transcription via recruiting EZH2 to TSC2 promoter regions. This study provides insight into a novel autophagy-associated mechanism whereby MALAT1 participates in AMI development.

## Data Availability

The datasets used and/or analysed during the current study are available from the corresponding author on reasonable request.

## Abbreviations

AMI: acute myocardial infarction
ANOVA: one-way analysis of variance
ChIP: chromatin immunoprecipitation
DMEM/F12: Dulbecco’s modified Eagle medium/F-12
EZH2: enhancer of zeste 2 polycomb repressive complex 2 subunit
FBS: fetal bovine serum
GFP: green fluorescent protein
H/R: hypoxia/reoxygenation
HRP: horseradish peroxidase
lncRNAs: m long non-coding RNAs
MALAT1: metastasis-associated lung adenocarcinoma transcript 1
mTOR: mammalian target of rapamycin
p-mTOR: phosphorylation level of mTOR
qRT-PCR: quantitative real-time PCR
RIP: RNA immunoprecipitation
SD: standard deviation
TSC2: tuberous sclerosis 2
HIF-1α: hypoxia inducible factor-1α
LDH: lactate dehydrogenase
